# Learning Motion-Induced Channel Dynamics with Multiresolution Multiplex Graphs for Wi-Fi CSI-Based Human Activity Sensing

**DOI:** 10.3390/s26185961

**Published:** 2026-09-20

**Authors:** Ying Xiao, Lin Li, Haiyong Zheng

**Affiliations:** 1 College of Electronic Engineering, Ocean University of China, Qingdao 266404, China; xiao.ying@wustl.edu (Y.X.); eelilin@ouc.edu.cn (L.L.); 2 Shandong Key Laboratory of Intelligent Sensing Chips and Systems, Ocean University of China, Qingdao 266404, China; 3 Shenzhen Research Institute, Ocean University of China, Shenzhen 518100, China

**Keywords:** Wi-Fi sensing, channel state information, human activity sensing, multiresolution analysis, multiplex graph neural network, signed graph learning, cross-condition recognition

## Abstract

**Highlights:**

**What are the main findings?**

- Spectral alignment and relative channel-response fields reduce acquisition-dependent input mismatch.
- Multiresolution multiplex graphs encode signed subcarrier relations within and across temporal resolutions.

**What is the implication of the main finding?**

- Signal constraints and relational inductive biases are learned within one CSI sensing framework.
- The measured complete model has a cross-domain mean of 48.81%, compared with 43.97% for the strongest of ten locally reevaluated baselines.

**Abstract:**

Wi-Fi channel state information (CSI) supports human activity recognition from motion-induced changes in indoor propagation, yet device, environment, and user changes perturb both channel responses and subcarrier relations. We present a model that aligns valid subcarriers across inputs and combines CSI response features with a multiresolution graph. Temporal decomposition produces residual components at multiple resolutions and a smooth component. The graph propagates information along positive and negative relations between subcarriers within each resolution and uses signal energy to guide propagation across resolutions. A gate incorporates the graph representation into the response classifier. A controlled multipath study further tests the physical interpretation of signed amplitude correlations. Experiments on CSI-Bench yield weighted-F1 scores of 95.96%, 50.16%, 44.08%, and 52.19% on the four protocols, with a cross-domain mean of 48.81%, exceeding the strongest of ten locally reevaluated baselines by 4.84 points.

## 1. Introduction

Human activities perturb indoor wireless propagation through reflection, diffraction, and scattering. In an orthogonal frequency-division multiplexing Wi-Fi link, these perturbations appear as time-varying channel state information (CSI) across multiple subcarriers, providing a signal basis for human activity recognition (HAR) with Wi-Fi infrastructure [1,2].

**Figure 1:**
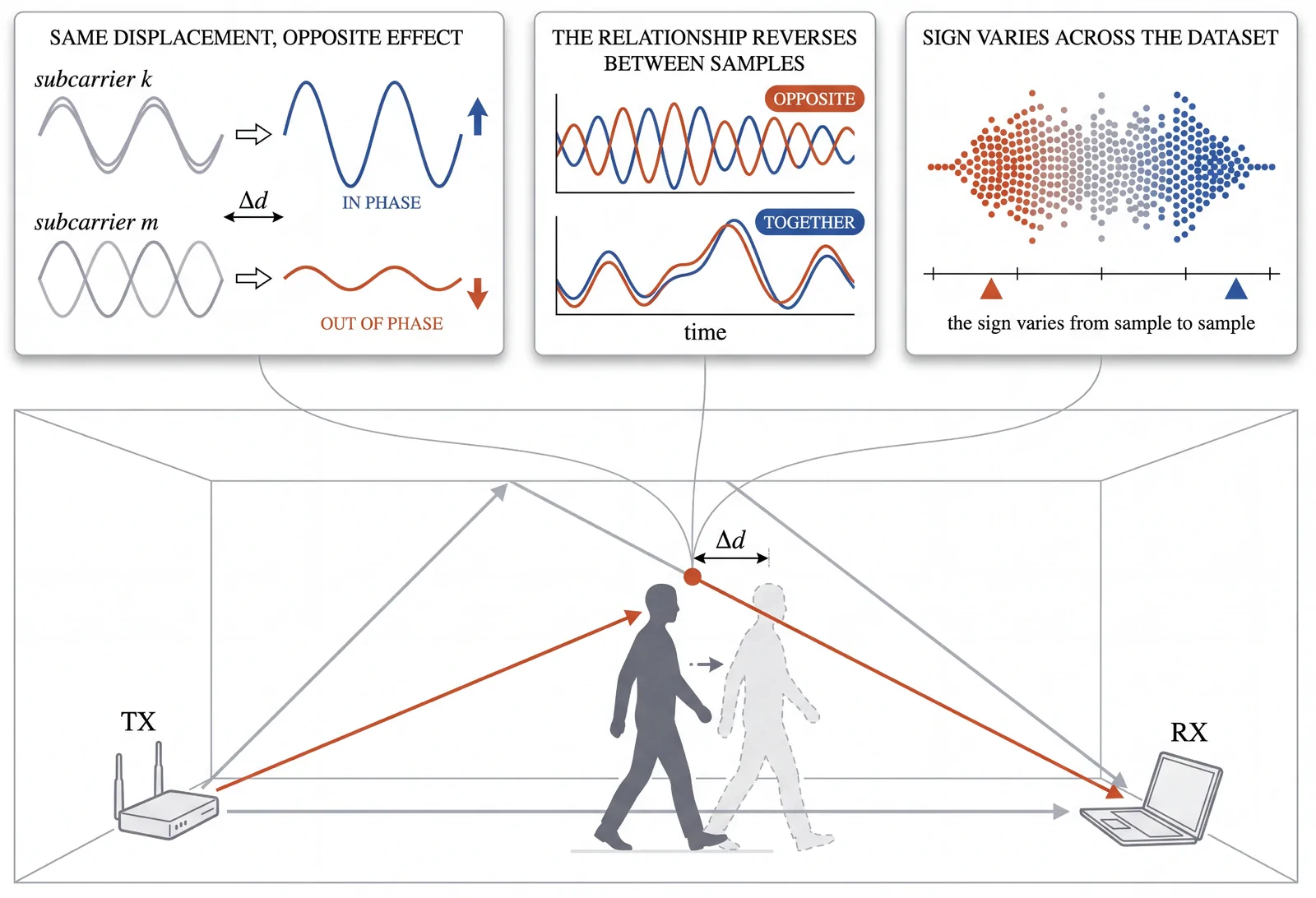
Motion-induced signed subcarrier dynamics in Wi-Fi CSI. A common displacement Δd perturbs frequency-selective multipath responses differently, producing co-varying or counter-varying subcarrier trajectories. As the propagation geometry changes across observations, the sign of a subcarrier relation becomes sample dependent, as depicted schematically in the right panel. In the room diagram, the gray arrows denote static propagation paths, the orange-red arrows denote the paths affected by the human displacement Δd, and the orange-red dot marks the moving reflection point. In the right panel, the orange-red and blue dots denote samples whose subcarrier relation is counter-varying and co-varying, respectively, and the triangles of the same colors mark the corresponding sample means.

Cross-condition CSI recognition presents two coupled representation challenges. (1) Devices expose different valid subcarrier intervals, so a fixed input index does not denote a consistent spectral coordinate. (2) Frequency-selective multipath produces sample-dependent co-varying and counter-varying subcarrier trajectories whose relational structure changes with acquisition conditions. Device, environment, and user changes therefore perturb both the response field and the relations through which motion is observed. Figure 1 illustrates the second effect in a controlled complex channel.

Existing CSI models encode packet sequences or image-like channel fields, but they usually receive a fixed subcarrier grid and model subcarrier dependence implicitly through convolution or attention. Field encoders learn local response patterns, while temporal models emphasize packet evolution; neither operation defines a common spectral coordinate together with an explicit sample-conditioned relation topology. Target-assisted methods address the resulting shift with deployment-condition data: M-FUDA [3] uses unlabeled target observations, whereas TCN-MAML [4] uses labeled target examples.

We address these challenges through complementary response and relational representations. Valid subcarriers are resampled to a common normalized coordinate, after which relative-amplitude, normalized-response, and temporal-change fields expose channel evidence. In parallel, constrained temporal analysis forms nested residual components, and a multiplex graph learns signed subcarrier propagation within resolutions and energy-conditioned transport across resolutions.

The network follows a unified hierarchy: spectral resampling establishes shared coordinates; the response encoder and multiresolution graph encoder learn complementary representations; and a sample-conditioned gate transports graph evidence to the response representation before activity classification. A controlled complex-channel study separately tests when signed amplitude correlations agree with phase-conditioned local sensitivity.

The contributions of this work are as follows:

1. We formulate a spectrally aligned CSI response representation and a constrained temporal analysis whose additive identity is defined on the rectified log-compressed field.
2. We formulate a multiresolution multiplex graph with sample-conditioned signed intra-resolution propagation, energy-conditioned inter-resolution transport, and gated graph-to-response conditioning. A controlled complex-multipath study validates the physical plausibility of the signed relation prior.
3. We reevaluate the seven architectures released with CSI-Bench and three baselines for domain adaptation and domain generalization using the same CSI-Bench data splits and preprocessing. The baselines share the training settings used for the response encoder. We select checkpoints using weighted-F1 on the source validation set and report the mean and standard deviation across three random seeds for each evaluation protocol.

The remainder of this paper is organized as follows. Section 2 reviews Wi-Fi CSI-based human activity sensing, deep learning for CSI representation, graph learning for wireless sensing, and cross-condition Wi-Fi sensing. Section 3 develops the constrained multiresolution analysis and multiplex graph formulation. Section 4 reports the evaluation protocol and results; Section 5 discusses the learned signal and graph structures; and Section 6 concludes the paper.

## 2. Related Work

### 2.1. Wi-Fi CSI-Based Human Activity Sensing

Wi-Fi sensing observes human motion through its perturbation of multipath propagation. Channel state information (CSI) resolves these perturbations across orthogonal frequency-division multiplexing subcarriers and time, providing finer propagation measurements than received signal strength [5,6]. This signal basis supports human activity recognition (HAR), gesture recognition, and identity inference, but these tasks use different label semantics and evaluation assumptions [7,8]. For HAR, CARM relates CSI dynamics to motion speed and activity profiles [9].

Evaluation has progressed from matched-condition datasets to cross-condition protocols. WiAR provides multiple activities, subjects, and acquisition factors for evaluating classifiers and deep models [10], whereas CSI-Bench standardizes Wi-Fi sensing tasks over heterogeneous devices, environments, and users [2]. These protocols test different properties: an in-domain split measures recognition under shared acquisition support, while cross-device, cross-environment, and cross-user splits test whether activity representations survive acquisition changes. Device changes alter the transceiver response and subcarrier layout, environment changes alter static multipath, and user changes alter the kinematics that generate activity evidence. Results are comparable when task labels, split construction, target information, and metrics coincide.

### 2.2. Deep Learning for CSI Representation

Deep CSI representations differ in the geometry imposed on channel dynamics. Sequence models capture packet-wise evolution through recurrent architectures or dilated temporal convolution, as exemplified by ABLSTM, STC-NLSTMNet, and WiFi-TCN [11,12,13]. Field-based models, including ImgFi and DF-CNN, transform CSI into spatial, angular, or channel-wise representations for convolutional processing [14,15]. Attention-based models such as THAT and WiTransformer learn long-range interactions across time and subcarriers [16,17]. These families emphasize temporal evolution, local structure, and long-range interaction. Local and global features are also combined in agricultural sensing and vision [18,19]. Temporal decomposition is also used in forecasting [20].

Other representation methods use multiscale analysis or self-supervised objectives. TCN-MAML applies fixed filtering and discrete wavelet decomposition before temporal convolution [4]; PA-CSI learns multiscale amplitude–phase features through convolution and attention [21]; and CAPC uses predictive coding and augmentation consistency to learn transferable CSI features [1]. These methods improve temporal feature learning, but variable valid-subcarrier support still leaves device-dependent spectral coordinates unresolved. Our response representation addresses that coordinate mismatch before feature encoding.

### 2.3. Graph Learning for Wireless Sensing

CSI encoders often model subcarrier dependence implicitly through their network operations. Convolution assumes local interactions on a regular index grid, whereas dense attention learns pairwise interactions without a channel topology [16,17]. Channel-wise models such as DF-CNN process CSI streams independently and postpone their interaction to decision fusion [15]. These mechanisms encode subcarrier dependence in convolution, attention, or fusion weights rather than in a graph topology.

Graph learning represents these relations through fixed or learned edges. Graph convolution and graph attention define propagation operators [22,23]. In Wi-Fi sensing, GrapHAR represents subcarriers as graph nodes [24]; graph few-shot learning constructs relational graphs among CSI activity embeddings [25]; and graph classification operates on time–frequency CSI features [26]. These CSI graphs model correlation, attention, or sample affinity. Signed graph convolution distinguishes positive and negative edges because they define different propagation semantics [27]. This leaves the problem of coupling sample-dependent signed subcarrier relations within each temporal resolution and corresponding subcarriers across resolutions.

### 2.4. Cross-Condition Wi-Fi Sensing

Source-only methods learn activity representations that transfer without access to target-condition observations. Widar3.0 derives body-coordinate velocity profiles for cross-domain gesture recognition, SHARP extracts Doppler features for person- and environment-independent HAR, and AirFi learns environment-general gesture features [28,29,30]. Wi-Cro and multi-source domain generalization increase source diversity through cross-domain generation or multi-source learning [31,32]. These methods address acquisition shifts through motion-oriented or environment-general representations and diversified source training.

Target-aware methods adapt the representation or decision process using information from the deployment condition. CrossFi supports zero- and few-shot recognition through similarity learning, whereas CDFi and AdaWiFi exploit limited target support for cross-domain or cross-environment adaptation [33,34,35]. M-FUDA aligns source features with unlabeled target distributions [3]; TCN-MAML adapts from labeled support samples [4]; and MDTA updates activity representations during test time [36]. Their information requirements span similarity-based inference, target samples, target distributions, and test-time parameter updates.

Cross-condition methods use motion features, source diversification, similarity learning, distribution alignment, or adaptation. The present work follows the source-only setting and jointly addresses two representation structures: heterogeneous valid-subcarrier support is aligned before response encoding, and sample-dependent signed relations are propagated within and across learned temporal resolutions.

## 3. Materials and Methods

### 3.1. Problem Formulation

For one sensing interval, let $X=\left[x_{t,f}\right]\in R^{T\times F}$ denote the CSI amplitude, where t∈{1,…,T} indexes packets and f∈{1,…,F} indexes subcarriers. Given *N* labeled observations and *C* activity classes, HAR is formulated as

(1) $$D={\left\{(X_{n},y_{n})\right\}}_{n=1}^{N},\;y_{n}\in \left\{1,\ldots ,C\right\},\;f_{\theta }:R^{T\times F}\to R^{C},$$

where θ contains the model parameters. The network estimates activity evidence from channel variation while limiting sensitivity to acquisition responses.

### 3.2. Method Overview

#### 3.2.1. Input Representation

Each observation contains a sample-specific valid subcarrier interval of width $F_{n}$. The loader first standardizes the valid amplitude samples. Spectral resampling then maps that interval to a common width $F_{c}$ and standardizes the interpolated field:

(2) $$X_{n}^{s}=\mathrm{Std}\left(X_{n}\right),\;{\bar{X}}_{n}=\mathrm{Std}\;\left(\mathrm{GridSample}(X_{n}^{s};F_{n},F_{c})\right)\in R^{T\times F_{c}},$$

where Std subtracts the sample mean and divides by the sample standard deviation. Both encoders operate on ${\bar{X}}_{n}$, so device-specific subcarrier counts do not change the network dimensions.

#### 3.2.2. Complementary Response and Graph Encoders

The response encoder $E_{r}$ applies learned coordinate alignment and residual encoding to relative-amplitude, normalized-response, and temporal-difference fields. The graph encoder $E_{g}$ analyzes a motion reference, *L* residual layers, and a final smooth layer. With $d_{e}$ denoting the representation dimension, their outputs are

(3) $$q_{n}=E_{r}\;\left(V_{r}\left({\bar{X}}_{n}\right)\right)\in R^{d_{e}},\;g_{n}=E_{g}\;\left(V_{g}\left({\bar{X}}_{n}\right)\right)\in R^{d_{e}}.$$

Human motion changes both the temporal content of CSI and the relations among subcarriers. The graph encoder represents these effects with a multiplex graph whose layers correspond to temporal resolutions. Let V be the shared spectral-node set and S={0,…,L+1} the layer set, where s=0 denotes the motion reference, s=1,…,L denote detail layers, and s=L+1 denotes the final smooth layer. The graph is

(4) $$G\left(X\right)=\left(V\times S,\;E_{\mathrm{intra}}\left(X\right)\cup E_{\mathrm{inter}}\left(X\right),\;{\left\{H_{s}\right\}}_{s\in S}\right),$$

where $H_{s}$ is the state of layer *s*, $E_{\mathrm{intra}}$ contains subcarrier edges within a layer, and $E_{\mathrm{inter}}$ connects corresponding nodes across layers.

#### 3.2.3. Prediction Hierarchy

**Figure 2:**
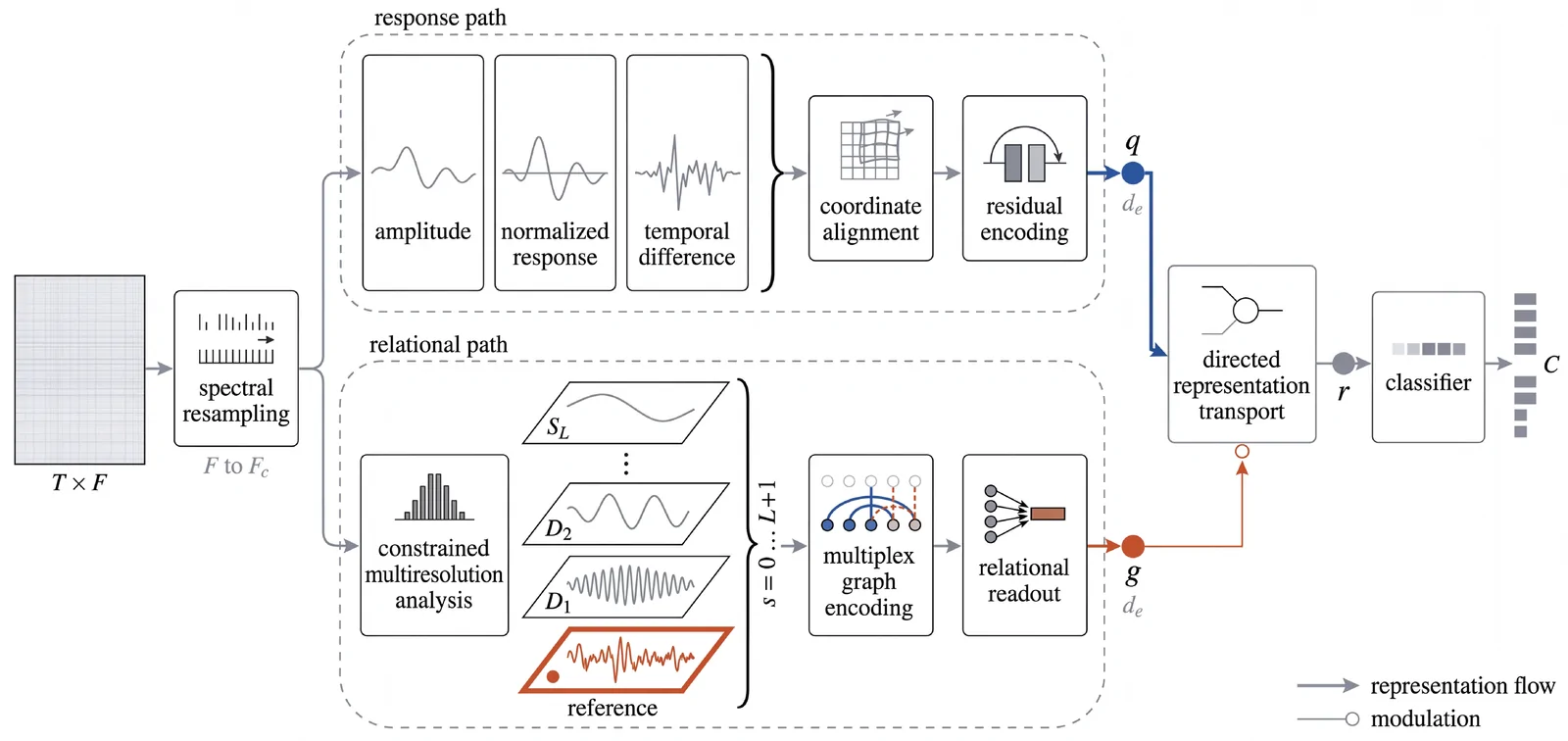
Architecture of the complete Wi-Fi CSI activity-sensing model. Spectral resampling maps the input to a common subcarrier coordinate. The response path encodes relative amplitude, normalized response, and temporal differences after learned coordinate alignment. The graph path applies constrained temporal analysis and multiplex propagation over signed intra-resolution and energy-conditioned inter-resolution relations. Gated conditioning transfers graph evidence to the response representation before classification. As indicated in the legend, the gray arrows denote representation flow and the line ending in an open circle denotes modulation; blue marks the response path, orange-red marks the relational path, and the framed blocks denote processing modules.

The classifier operates on a response-anchored representation conditioned by the graph embedding:

(5) $$X_{n}\overset{\mathrm{resampling}}{\to }{\bar{X}}_{n}\overset{E_{r},E_{g}}{\to }\left(q_{n},g_{n}\right)\overset{C}{\to }r_{n}\overset{\psi_{\mathrm{cls}}}{\to }\ell_{n},$$

where C denotes the gated relational conditioning defined in Section 3.7. Figure 2 summarizes the two paths and their response-anchored integration.

### 3.3. Constrained Multiresolution Relation Analysis

#### 3.3.1. Symmetric Temporal Analysis

Human activities combine transient, periodic, and sustained motion. The graph path therefore uses learnable nested smoothing to expose complementary temporal residual structure.

The input has already been standardized and contains positive and negative values. The temporal analysis retains its positive deviations and compresses their range:

(6) $$Z\left(t,f\right)=\log\;\left(1+\max\{\bar{X}\left(t,f\right),0\}\right),$$

which defines a rectified log-compressed representation. Values below the sample mean are mapped to zero; reconstruction claims below therefore apply to Z, not to raw or standardized CSI.

Temporal smoothing should not introduce a preferred time direction or change a constant channel response. At resolution l∈{1,…,L}, we therefore constrain a radius-*R* kernel to the symmetric probability simplex

(7) $$\begin{array}{cc} \Delta_{R}^{\mathrm{sym}} & =\left\{h\in R^{2R+1}\;:\;h\left(-r\right)=h\left(r\right),\;h\left(r\right)\geq 0,\;\sum_{r=-R}^{R}h\left(r\right)=1\right\},\\ q_{l,r} & =\frac{\exp(a_{l,r})}{\sum_{u=0}^{R}\exp\left(a_{l,u}\right)},\;h_{l}\left(0\right)=q_{l,0},\;h_{l}\left(\pm r\right)=\frac{1}{2}q_{l,r}\;\left(1\leq r\leq R\right), \end{array}$$

where ${\left\{a_{l,r}\right\}}_{r=0}^{R}$ are unconstrained trainable parameters. Thus, $h_{l}\in \Delta_{R}^{\mathrm{sym}}$ by construction. Symmetry removes directional temporal bias, while nonnegativity and unit mass make each smooth component a convex temporal average with unit DC gain.

#### 3.3.2. Additive Multiresolution Decomposition

A smooth response alone would discard the temporal variation that carries motion. We retain the removed content as a complementary detail. Starting with $S_{0}=Z$, recursive analysis defines

(8) $$S_{l}=h_{l}{*}_{t}S_{l-1},\;D_{l}=S_{l-1}-S_{l},\;l=1,\ldots ,L,$$

where ${*}_{t}$ denotes temporal convolution. The detail $D_{l}$ is the content removed by smoothing at level *l*.

The residual definition gives the additive identity

(9) $$Z=\sum_{l=1}^{L}D_{l}+S_{L}.$$

Because the intermediate smooth terms cancel. This identity is an implementation property rather than evidence of spectral separation. The constraints in Equation (7) define nested zero-phase smoothers; their usefulness is assessed empirically.

#### 3.3.3. Motion-Resolved Signal Fields

The scalar components in Equation (9) reconstruct Z but do not expose the local changes needed for relation analysis. We therefore lift each component to a multichannel field. A residual layer contains variance-normalized amplitude, temporal difference, and local motion energy. The smooth layer contains amplitude, temporal difference, and spectral difference. A reference field applies temporal normalization to $\bar{X}$ and retains its temporal difference and local energy.

### 3.4. Channel-Response Representation

#### 3.4.1. Response Field Construction

The response path retains relative channel structure after sample standardization. From the resampled field $\bar{X}$, we construct

(10) $$A={\mathrm{Norm}}_{t,f}\;\left[\bar{X}\;∥\;\frac{\bar{X}-\mu_{t}\left(\bar{X}\right)}{\sqrt{{\mathrm{Var}}_{t}\left(\bar{X}\right)+\delta }}\;∥\;\Delta_{t}\bar{X}\right],$$

where ${\mathrm{Norm}}_{t,f}$ scales each field by its temporal–spectral standard deviation. The constituent fields encode relative amplitude, temporally normalized response, and packet-to-packet change; they do not retain absolute receiver gain.

#### 3.4.2. Deformable Coordinate Alignment

Packet timing and subcarrier responses vary across acquisitions, so the response path aligns its input before residual encoding. Following differentiable coordinate sampling in spatial transformer networks [37], a convolutional context network predicts a temporal–spectral offset $\Omega_{\phi }$ and an attention gate $M_{\phi }$. With W denoting bilinear sampling, the aligned field is

(11) $$\begin{array}{cc} \tilde{A} & =W\;\left(A,G_{0}+\Omega_{\phi }\left(A\right)\right),\\ A^{\prime } & =A+M_{\phi }\left(A\right)⊙\left(\tilde{A}-A\right)+\Phi_{\phi }\left(\left[A∥\tilde{A}\right]\right), \end{array}$$

where $G_{0}$ is the regular sampling grid and $\Phi_{\phi }$ refines the aligned channels. Both offset and gate predictions are conditioned on pooled CSI context.

#### 3.4.3. Residual Response Encoding

The aligned field is passed to a residual encoder [38]. Stacked residual transformations preserve local channel structure while expanding the receptive field; global pooling produces the response embedding

(12) $$q=E_{r}\left(A^{\prime }\right)\in R^{d_{e}}.$$

### 3.5. Multiresolution Multiplex Graph Formulation

#### 3.5.1. Resolution-Shared Spectral Nodes

The sensing fields use the same temporal–spectral coordinates and graph nodes. The coordinate-alignment operator in Equation (11) and a convolutional stem are shared across fields. The stem maps field *s* to $H_{s}\in R^{d_{v}\times T^{\prime }\times M}$, where $d_{v}$ is the node-state dimension and *M* is the number of spectral nodes. The slice $H_{s,i}=H_{s,:,:,i}\in R^{d_{v}\times T^{\prime }}$ is the trajectory of node i∈V={1,…,M}. Node *i* denotes the same subcarrier neighborhood in every resolution layer. All quantities in this subsection describe one graph stage; stage indices are restored in Section 3.6.

#### 3.5.2. Signed Intra-Resolution Relations

Fixed subcarrier indices do not describe sample-specific motion relations. We therefore infer graph edges from the temporal mean, standard deviation, and mean absolute change of each reference node:

(13) $$e_{i}=\phi_{n}\;\left(\left[\mu_{t}\left(H_{0,i}\right)\;∥\;\sigma_{t}\left(H_{0,i}\right)\;∥\;\mu_{t}\left(\right|\Delta_{t}H_{0,i}\left|\right)\right]\right).$$

Here, $\phi_{n}$ is a learned node embedding, while $\mu_{t}$, $\sigma_{t}$, and $\Delta_{t}$ denote the temporal mean, standard deviation, and first difference, respectively. The descriptor $e_{i}$ is used only to infer edges; the full trajectory $H_{0,i}$ remains available for message passing.

Multipath propagation can make two subcarriers vary in the same or in opposite directions. A non-negative graph cannot distinguish these cases. Let $m_{i}$ be the channel-averaged, temporally centered trajectory of reference node *i*. We retain the *K* strongest absolute correlations and preserve their signs:

(14) $$\begin{array}{cc} \rho_{ij} & =\frac{\langle m_{i},m_{j}\rangle }{{∥m_{i}∥}_{2}{∥m_{j}∥}_{2}+\delta },\;N_{i}^{\pm }={\mathrm{TopK}}_{j\neq i}\left|\rho_{ij}\right|,\\ A_{ij}^{\pm } & =\frac{\rho_{ij}\;1\left\{j\in N_{i}^{\pm }\right\}}{\sum_{u\in N_{i}^{\pm }}\left|\rho_{iu}\right|+\delta }, \end{array}$$

where 1{·} is the indicator function and δ>0 is a numerical stabilizer. Positive and negative entries of $A_{ij}^{\pm }$ represent co-varying and counter-varying trajectories, respectively. Normalization by absolute degree retains the observed correlation sign while controlling message magnitude.

Correlation provides a signed relation prior, but it does not determine which learned features should exchange information. We therefore combine it with a sparse learned topology. For attention head *h*,

(15) $$\begin{array}{cc} b_{ij}^{h} & =\frac{\langle W_{q}^{h}e_{i},W_{k}^{h}e_{j}\rangle }{\sqrt{d_{h}}}+\eta \rho_{ij},\;N_{i}^{+,h}={\mathrm{TopK}}_{j}b_{ij}^{h},\\ A_{ij}^{+,h} & =\frac{\exp\left(b_{ij}^{h}\right)\;1\left\{j\in N_{i}^{+,h}\right\}}{\sum_{u\in N_{i}^{+,h}}\exp\left(b_{iu}^{h}\right)}. \end{array}$$

Here, $d_{h}$ is the head dimension and η weights the correlation prior. The learned matrix $A^{+,h}$ transports compatible features, whereas $A^{\pm }$ retains the observed correlation sign. Both are inferred from the reference and reused by the signal layers.

#### 3.5.3. Energy-Conditioned Inter-Resolution Relations

The two intralayer graphs leave the temporal resolutions disconnected, although one activity may contain both fast and slow motion. We therefore connect the layers using feature compatibility and their relative energy. With $\mu_{t,i}$ denoting the mean over temporal and node axes, define

(16) $$\begin{array}{cc} g_{s} & =\mu_{t,i}\;\left(\mathrm{GN}(H_{s})\right),\;E_{s}=\frac{∥H_{s}{∥}_{F}^{2}}{d_{v}T^{\prime }M},\\ {\tilde{E}}_{s} & =\log\left(E_{s}+\delta \right)-\frac{1}{\left|S\right|}\sum_{u\in S}\log\left(E_{u}+\delta \right),\\ P_{rs} & ={\mathrm{softmax}}_{s}\;\left(\frac{\langle W_{q}^{g}g_{r},W_{k}^{g}g_{s}\rangle }{\sqrt{d_{g}}}+\beta {\tilde{E}}_{s}\right). \end{array}$$

**Figure 3:**
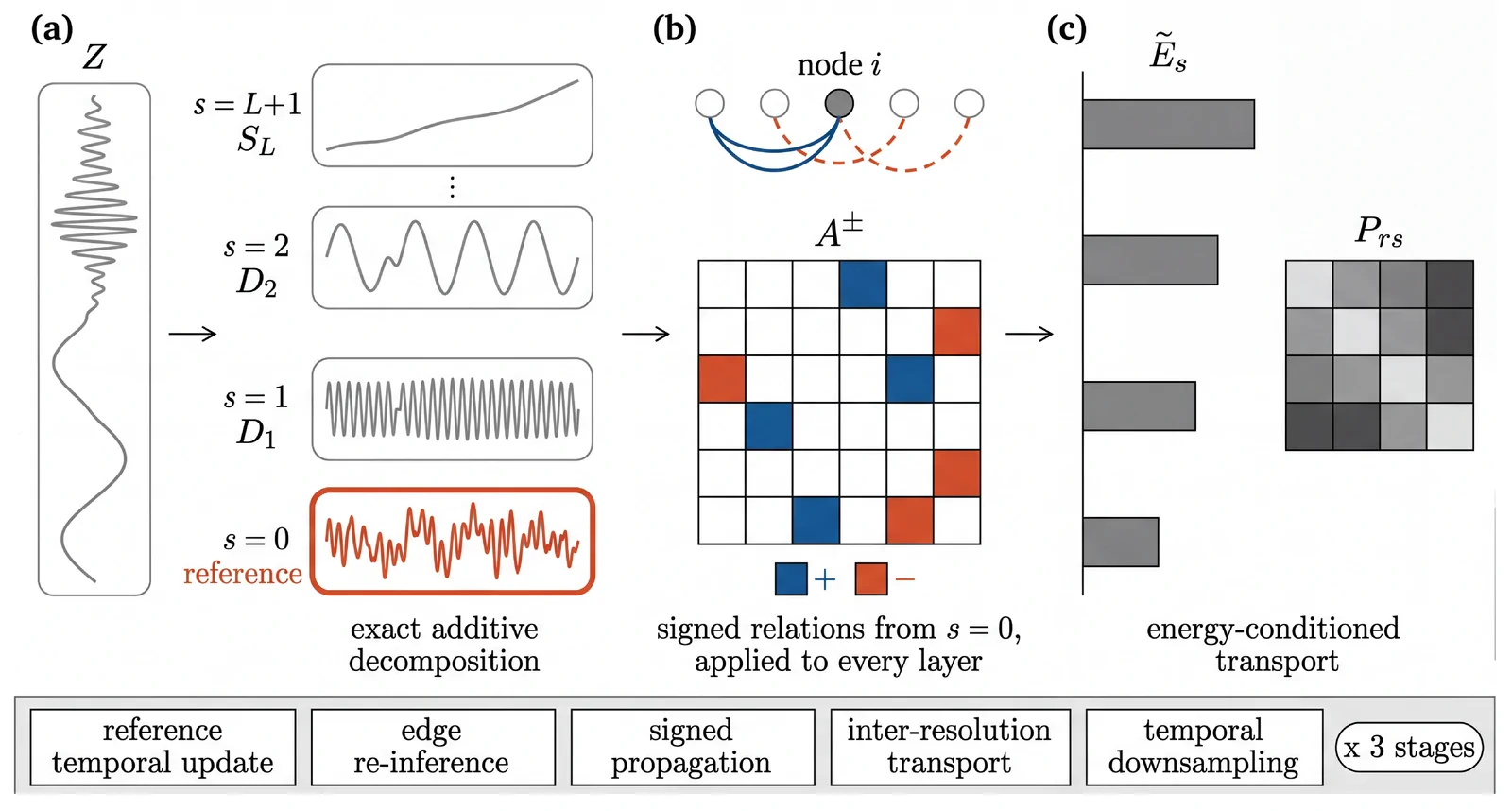
Auxiliary multiplex graph construction. (**a**) The rectified log-compressed field Z is expressed as nested smoothing residuals and a final smooth component following Equation (8). (**b**) Sample-conditioned signed relations $A^{\pm }$ are inferred from the reference layer—blue and orange-red cells denote co-varying and counter-varying trajectories, and the empty diagonal reflects the exclusion of self-relations in Equation (14)—and reused by every signal layer through Equation (15). (**c**) Inter-resolution weights $P_{rs}$ combine feature compatibility with centered log-energy ${\tilde{E}}_{s}$. The bottom strip lists the operations within each graph stage. In (**b**), the gray filled dot denotes the current node *i* and the white dots denote the other spectral nodes, while blue solid and orange-red dashed edges denote positive and negative relations, respectively.

Here, GN denotes group normalization, ${\tilde{E}}_{s}$ is the centered log-energy, $d_{g}$ is the descriptor dimension, and β controls the energy term. The softmax makes each row of P sum to one. Thus, a layer receives more information from resolutions that are both feature-compatible and active in the observation. Figure 3 summarizes the additive residual layers, the signed relations inferred once from the reference and shared by all layers, and the energy-conditioned transport between resolutions.

### 3.6. Multiplex Graph Representation Learning

#### 3.6.1. Multistage Graph Propagation

The graph construction above specifies the nodes and edges. The encoder propagates features through *B* graph stages indexed by k∈{0,…,B−1}. At each stage, the reference is updated in time and used to infer $A^{\pm ,(k)}$ and $A^{+,h,(k)}$. These matrices propagate the signal layers, after which $P^{\left(k\right)}$ transports information across resolutions. Temporal downsampling between selected stages expands the effective temporal context while preserving the node-state and spectral-node axes.

#### 3.6.2. Multiscale Temporal Dynamics

Graph edges describe relations among subcarriers, but activity recognition also requires the temporal evolution of each node. We therefore update the reference with gated depthwise convolutions over a dilation set Q:

(17) $$\begin{array}{cc} {\tilde{H}}_{0}^{\left(k\right)} & =H_{0}^{\left(k\right)}+G_{0}^{\left(k\right)}⊙\Phi_{t}^{\left(k\right)}\;\left(\underset{q\in Q}{∥}{\mathrm{DWConv}}_{q}\left(\mathrm{GN}\left(H_{0}^{\left(k\right)}\right)\right)\right),\\ G_{0}^{\left(k\right)} & =\sigma \;\left(W_{g}^{\left(k\right)}\mathrm{GN}\left(H_{0}^{\left(k\right)}\right)\right), \end{array}$$

where ${\mathrm{DWConv}}_{q}$ is a depthwise temporal convolution with dilation *q*, $\Phi_{t}^{\left(k\right)}$ fuses the multiscale responses, and $G_{0}^{\left(k\right)}$ is a pointwise gate. The convolutions act only along time and therefore preserve the spectral nodes. The graph operators in Equations (14) and (15), followed by pointwise channel mixing, map ${\tilde{H}}_{0}^{\left(k\right)}$ to the updated reference ${\hat{H}}_{0}^{\left(k\right)}$.

#### 3.6.3. Signed Intra-Resolution Propagation

The reference supplies a motion topology shared by the signal layers, whose temporal content differs. Let $V_{s}^{h,(k)}$ be the head-wise values obtained from short- and long-range temporal filters. For s>0,

(18) $$\begin{array}{cc} M_{s}^{\left(k\right)} & =\underset{h}{∥}\left(A^{+,h,(k)}V_{s}^{h,(k)}+\lambda A^{\pm ,(k)}V_{s}^{h,(k)}\right),\\ {\hat{H}}_{s}^{\left(k\right)} & =H_{s}^{\left(k\right)}+\tanh\left(\kappa^{\left(k\right)}\right)\;\Phi_{g}^{\left(k\right)}\left(M_{s}^{\left(k\right)}\right),\;s\in S\setminus \left\{0\right\}, \end{array}$$

where λ balances the signed message, $\Phi_{g}^{\left(k\right)}$ projects the concatenated heads, and $\tanh(\kappa^{\left(k\right)})$ gates the residual update. The first message transports compatible features; the second adds or subtracts a neighbor according to the observed correlation sign.

#### 3.6.4. Inter-Resolution Relational Coupling

Intralayer propagation models subcarrier relations at one resolution, but it does not combine evidence across resolutions. We therefore apply the transport matrix $P^{\left(k\right)}$ after every graph update. With $W_{v}^{g,(k)}$ shared across layers, the update for r∈S is

(19) $$H_{r}^{\left(k+1\right)}={\hat{H}}_{r}^{\left(k\right)}+\tanh\left(\gamma^{\left(k\right)}\right)W_{o}^{\left(k\right)}\left[\sum_{s\in S}P_{rs}^{\left(k\right)}W_{v}^{g,(k)}\mathrm{GN}\left({\hat{H}}_{s}^{\left(k\right)}\right)-W_{v}^{g,(k)}\mathrm{GN}\left({\hat{H}}_{r}^{\left(k\right)}\right)\right].$$

The scalar $\tanh(\gamma^{\left(k\right)})$ gates the residual, and $W_{o}^{\left(k\right)}$ projects it back to the layer state. Subtracting the self-value makes the update depend on transported context relative to the layer state.

#### 3.6.5. Relational Readout

The transport module requires one fixed-length graph vector, whereas the encoder ends with several graph layers. We summarize each final layer using attention-weighted, mean, maximum, and standard-deviation pooling. Let $u_{s}$ denote the layer representation, $\Phi_{p}$ and $\Phi_{r}$ learned projections, and LN layer normalization. The readout is

(20) $$\begin{array}{cc} u_{s} & =\Phi_{p}\;\left([\mathrm{AttPool}\left(H_{s}^{\left(B\right)}\right)∥\mu \left(H_{s}^{\left(B\right)}\right)∥\max\left(H_{s}^{\left(B\right)}\right)∥\sigma \left(H_{s}^{\left(B\right)}\right)]\right),\\ \omega_{s} & ={\mathrm{softmax}}_{s}\;\left(\mathrm{Score}(\mathrm{LN}\left(u_{s}\right))\right),\;s\in S\setminus \left\{0\right\},\\ g & =u_{0}+\Phi_{r}\;\left(\left[u_{0}∥\sum_{s\in S\setminus \{0\}}\omega_{s}u_{s}\right]\right). \end{array}$$

The reference $u_{0}$ summarizes motion, while the weighted sum selects temporal-resolution evidence for the observation. Their combination yields the graph embedding $g\in R^{d_{e}}$.

### 3.7. Gated Relational Conditioning

#### 3.7.1. Graph-Conditioned Response Representation

The response embedding q carries channel-response evidence, while g carries temporal and subcarrier relations. A scalar compatibility gate uses the latter as a residual correction. Let $d_{a}$ be the affinity dimension. The gate, relational message, and conditioned representation are

(21) $$\begin{array}{cc} a_{n} & =\sigma \;\left(\frac{\langle W_{q}^{a}\mathrm{LN}\left(q_{n}\right),W_{k}^{a}\mathrm{LN}\left(g_{n}\right)\rangle }{\sqrt{d_{a}}}+b_{a}\right),\\ m_{n} & =\Phi_{a}\;\left(a_{n}W_{v}^{a}\mathrm{LN}\left(g_{n}\right)\right),\\ r_{n} & =q_{n}+\sigma \left(\zeta \right)m_{n}. \end{array}$$

Here, $\Phi_{a}$ is a learnable normalized projection and σ(ζ) controls the residual magnitude. The update retains the response embedding as the classifier anchor while adding a sample-conditioned graph message.

#### 3.7.2. Prediction and Learning Objective

A learnable classifier maps $r_{n}$ to activity logits:

(22) $$\begin{array}{cc} \ell_{n} & =\psi_{\mathrm{cls}}\left(\mathrm{LN}\left(r_{n}\right)\right),\\ p_{n,c} & =\frac{\exp(\ell_{n,c})}{\sum_{j=1}^{C}\exp\left(\ell_{n,j}\right)},\;{\hat{y}}_{n}=\arg\max_{c}p_{n,c}. \end{array}$$

The response and graph encoders remain fixed while relational conditioning is learned. Let $\ell_{n}^{r}$ denote the response-encoder logits, τ>0 the distillation temperature, and $\lambda_{\mathrm{KL}}\geq 0$ its weight [39]. The objective is

(23) $$L_{\mathrm{tr}}=\frac{1}{N}\sum_{n=1}^{N}\mathrm{CE}\left(\ell_{n},y_{n}\right)+\lambda_{\mathrm{KL}}\tau^{2}\mathrm{KL}\;\left(\mathrm{softmax}\left(\ell_{n}^{r}/\tau \right)\;∥\;\mathrm{softmax}\left(\ell_{n}/\tau \right)\right).$$

This stage optimizes the gated correction while leaving the two pretrained encoder representations fixed. The additive identity in Equation (9) follows directly from the residual definition and requires no auxiliary loss.

## 4. Results

### 4.1. Dataset and Evaluation Protocols

All methods are evaluated using the fixed CSI-Bench human activity recognition splits [2], with five classes: jumping, running, seated breathing, walking, and hand waving. The training and validation sets contain 15,843 and 3393 observations; in-domain (ID), cross-device, cross-environment, and cross-user testing use 3400, 4958, 5660, and 5967 observations, respectively. Table 1 lists their acquisition factors.

Weighted-F1 is the primary metric. Measured results are summarized across seeds 42, 2025, and 3407 using the mean and sample standard deviation. The cross-domain (OOD) mean weights the cross-device, cross-environment, and cross-user protocol scores equally.

### 4.2. Implementation and Baselines

Inputs contain at most 232 stored subcarriers and are resampled to $F_{c}=96$ spectral positions. The response encoder outputs a 512-dimensional representation, and its classifier has widths 512→256→5. The auxiliary analysis uses L=2, filter radius R=5, M=48 spectral nodes, K=12 neighbors, node width $d_{v}=128$, and three temporal dilations {1,2,4}. Its signed-message coefficient is λ=0.1. The source-screened auxiliary checkpoint uses a weight-interpolation coefficient ξ=0.3 between its neutral and learned endpoints; ξ is distinct from the trainable residual gates in Equations (18) and (19).

Optimization uses AdamW and batches of 64 observations. Each encoder is first trained with its own classifier under cross-entropy loss. The response model is trained for 18 epochs and refined for four epochs with device–user-balanced sampling; its learning rate in the refinement stage is $3\times 10^{-5}$. The graph encoder starts from a 12-epoch single-resolution model and trains its multiresolution modules for eight epochs at $5\times 10^{-5}$. Gated conditioning freezes both encoders and trains the gated correction for at most four epochs at $10^{-4}$. Checkpoints are selected using weighted-F1 on the source validation set. Training and evaluation use PyTorch 2.11 with CUDA; inference uses bfloat16 arithmetic on an NVIDIA GeForce RTX 4090 D.

We locally reevaluate the seven architectures released with CSI-Bench (LSTM, MLP, PatchTST, ResNet-18, TimeSformer-1D, Transformer, ViT) and three baselines for domain adaptation and domain generalization (CORAL, DANN, and SWAD). All methods use the same three random seeds. Checkpoints are selected using weighted-F1 on the source validation set, and all training stages are completed before evaluation on the four protocols.

CORAL, DANN, and SWAD use the Transformer architecture as their backbone. CORAL aligns source-domain covariance matrices with loss weight α=1.0, without target-domain observations. DANN applies adversarial domain alignment using source-domain device labels as the domain indicator; the gradient-reversal weight is linearly annealed from 0 to 1.0 over the first half of training. SWAD averages model weights along the training trajectory; averaging begins after the validation loss first increases and ends at convergence, following the dense stochastic weight averaging protocol of Cha et al. (2021) [40]. All three baselines are evaluated in the source-only setting.

The shared input consists of standardized CSI features at 96 subcarrier positions, represented by three response fields. Sequence models use 288 concatenated features; ResNet-18 and ViT use three channels resized to 224×224, matching the response CNN. The proposed model retains its learned alignment and graph operations. The dropout rates are 0.3 for LSTM and 0.1 for Transformer and CORAL; ResNet-18 has no corresponding dropout.

Each baseline uses the same training schedule as the response encoder, with the same optimizer (AdamW, weight decay $10^{-4}$), learning rate schedule (cosine annealing from $3\times 10^{-4}$ to $10^{-6}$), data augmentation (Gaussian noise with σ=0.01 and random time masking of up to 10% of frames), class-balanced sampling, and batch size of 64. The complete model additionally trains its graph encoder and fusion module, so the total training computation differs across methods. All results in Table 2 are obtained from these local reruns.

### 4.3. Comparison with Existing Methods

Table 2 reports all ten locally reevaluated baselines and the complete model under the same evaluation protocols. The complete model obtains 95.96±0.08%, 50.16±0.44%, 44.08±0.46%, and 52.19±0.59% on ID, cross-device, cross-environment, and cross-user data, respectively. The protocol-specific margins over the strongest baseline in each column are 3.79, 7.79, 4.25, and 1.67 percentage points, averaging 4.38 points. The resulting OOD mean is 48.81±0.50%, 4.84 points above the strongest baseline OOD mean (LSTM, 43.97±1.18%). SWAD is strongest on ID data; LSTM leads cross-device, cross-environment, and OOD; Transformer leads cross-user.

#### Measured Effect of Graph Conditioning

Table 3 compares the response-only and complete models under the same protocols.

The measured complete-minus-response OOD difference is 1.80±0.06 percentage points, with positive differences in all three seeds. The mean differences for ID, cross-device, cross-environment, and cross-user evaluation are +0.15±0.00, +1.80±0.06, +1.50±0.08, and +2.10±0.05 points, respectively.

The response-only encoder already exceeds ResNet-18 by 8.29 OOD points (47.01% vs. 38.72%). This gap reflects the response encoder being designed for CSI data—learned subcarrier alignment maps non-uniform subcarrier positions to a regular grid, and spectral resampling constructs the input representation from the three response fields—whereas ResNet-18 applies generic 2D convolutions to the shared CSI input without these learned operations.

Table 4 reports complementary measured metrics from saved predictions.

**Table 4:** Complementary metrics on the four official test protocols. All values are percentages and are derived from the confusion matrices in Figure 4.

Protocol	Accuracy	Macro-F1	Weighted-F1
ID	95.97	94.46	95.96
Cross-device	50.79	41.03	50.16
Cross-environment	48.73	30.81	44.08
Cross-user	57.97	38.03	52.19

### 4.4. Class-Wise Recognition Under Acquisition Changes

The ID per-class recall ranges from 89.18% to 98.78%. Under device change, seated breathing retains 80.52% recall and walking retains 62.78%. Under environment and user changes, walking remains the most stable dynamic class, with recalls of 80.13% and 94.67%, respectively.

Most misclassified jumping and running samples are predicted as walking. This pattern motivates examining whether velocity profiles or Doppler features could improve recognition when the device, environment, or user changes.

The class-wise results distinguish activity-dependent effects hidden by aggregate metrics. Figure 5 shows that seated breathing obtains 72.20% F1 under device change, while walking obtains 66.32% and 75.45% under environment and user changes. These sustained-pattern classes are recognized more reliably than jumping, running, and hand waving. The measured class-balanced results are reported separately in Table 4.

### 4.5. Analysis of Signal and Graph Components

To evaluate the six components separately, we compare the complete model with six configurations, each removing or replacing one component while retaining the remaining structure. Without spectral resampling, 96 fixed, evenly spaced positions on the original subcarrier grid are selected from the input padded to 232 subcarrier positions; normalization after sampling and network width are retained. This control tests the use of the valid subcarrier interval relative to fixed sampling. Without coordinate alignment, the input alignment operation is replaced by the identity in both encoders. Without multiresolution decomposition, the same representation Z without temporal decomposition is supplied to the three branches that process the temporal components, retaining the motion reference, branch capacity, coupling, and readout. Without added signed propagation, the additional signed messages to these three branches are disabled while non-negative attention and the branch for the motion reference are retained. Without inter-resolution coupling, the inter-resolution coupling modules are replaced by identity mappings, retaining the branches and final readout. Without gated conditioning, the gate computed for each sample is fixed to one while the trainable residual correction is retained.

All six configurations use the same data splits and three random seeds as the complete model. Checkpoints are selected using weighted-F1 on the source validation set. We rerun the affected training stages for each configuration and report the mean OOD weighted-F1 over the three seeds.

Table 5 shows lower mean OOD weighted-F1 for all six ablated configurations. Removing spectral resampling reduces the score from 48.81% to 46.99%, the largest decrease of 1.82 percentage points. Removing multiresolution decomposition produces the next largest decrease, 1.39 points. These results support using both the valid subcarrier interval and multiple temporal resolutions when constructing the CSI representation.

Disabling added signed propagation or inter-resolution coupling reduces the score by 0.85 and 0.68 points, respectively, supporting the use of signed subcarrier relations and information exchange across resolutions. Removing coordinate alignment or fixing the gate to one produces smaller decreases of 0.46 and 0.44 points. For all six components, the paired complete-minus-ablation difference is positive at every seed (paired standard deviations range from 0.09 to 0.19 pp). The two largest decreases (spectral resampling and multiresolution decomposition) are well separated from zero; the four smaller decreases (0.44–0.85 pp) are directionally consistent across seeds but closer to the per-seed noise floor.

Figure 6 illustrates graph-path and graph-conditioning comparisons. The measured response-only comparison is given in Table 3.

Figure 7 examines the constrained temporal transform directly. Both kernels are symmetric and non-negative, and each has measured sum 1.000000. Their 0.5-magnitude crossings are 0.0957 and 0.0879 cycles/frame, defining nested smoothers with overlapping responses. These values are stable across training runs because the architecture constrains each kernel to be symmetric, non-negative, and unit-sum with a fixed radius, which limits the solution space. Summing the two residuals and the final smooth component yields mean and maximum numerical errors of $2.53\times 10^{-10}$ and $2.38\times 10^{-7}$, respectively, numerically verifying Equation (9).

The two smoothing kernels in Figure 7 have similar frequency responses. Recursive decomposition produces $D_{1}$ by subtracting the first smoothed representation from Z, $D_{2}$ by subtracting the second smoothed representation from the first, and $S_{2}$ as the final smoothed representation. Let $H_{1}\left(\omega \right)$ and $H_{2}\left(\omega \right)$ denote the smoothing responses. Away from the window boundaries, the effective component responses are

$$R_{D_{1}}=1-H_{1},\;R_{D_{2}}=H_{1}\left(1-H_{2}\right),\;R_{S_{2}}=H_{1}H_{2}.$$

Similar smoothing responses therefore do not imply identical responses for the three components. Their frequency responses can overlap while weighting temporal variations differently. Reflection padding is used at the window boundaries. The following experiments evaluate whether combining these components improves recognition.

Table 6 and Figure 8 compare the input without temporal decomposition, each component alone, all three pairs, and the complete combination of $D_{1}$, $D_{2}$, and $S_{2}$. All configurations retain the motion reference and the fusion of graph and response features. The complete model and the control without temporal decomposition correspond to those in Table 5.

The complete combination achieves 48.81% OOD weighted-F1, exceeding the input without temporal decomposition by 1.39 percentage points, the best single component ($S_{2}$, 48.05%) by 0.76 points, and the best pair ($D_{2}+S_{2}$, 48.51%) by 0.30 points. Adding $D_{1}$, $D_{2}$, or $S_{2}$ to the other two components produces gains of 0.30, 0.39, and 0.55 points, respectively.

All paired complete-minus-configuration differences are positive at every seed. The smallest difference (complete vs. best pair, +0.30±0.14 pp) is directionally consistent but close to the per-seed noise floor, providing limited evidence of complementarity for the third component; the larger differences (complete vs. undecomposed, +1.39±0.19 pp) are more clearly separated from zero. These gains support complementary contributions to activity recognition within the proposed model, even though the component frequency responses overlap.

### 4.6. Controlled Complex-Multipath Study

To test the interpretation of signed amplitude correlations, we generate a frequency-selective complex channel with a static term and one motion-dependent path:

(24) $$H_{f}\left(t\right)=H_{f}^{s}+a_{f}\exp\;\left[-j2\pi f\;\left(\tau_{f}+\frac{2d(t)}{c}\right)\right],\;x_{f}\left(t\right)=\left|H_{f}\left(t\right)\right|+\varepsilon_{f}\left(t\right),$$

where d(t) is a shared displacement and $\varepsilon_{f}\left(t\right)$ is additive noise. For small displacement, the local amplitude sensitivity $g_{f}=\partial \left|H_{f}\right|/\partial d$ predicts that two subcarriers should co-vary when $g_{i}g_{j}>0$ and counter-vary when $g_{i}g_{j}<0$. We compare this analytic sign with the empirical correlation sign used in Equation (14).

The CUDA PyTorch study, implemented in PyTorch 2.11.0 with CUDA 12.8, samples 512 channel realizations at each signal-to-noise ratio (SNR). Figure 9 shows sign-agreement rates of 69.1%, 94.1%, and 98.5% at 10, 20, and 30 dB, while approximately half of the selected relations are negative at every SNR. Thus, a complex multipath response can generate stable positive and negative amplitude relations when the measurements are sufficiently resolved. The experiment supports this mechanism as an interpretation of signed correlations; the learned graph still estimates statistical relations from amplitude trajectories.

The gate distributions in Figure 10 illustrate sample-dependent conditioning.

#### Signed Relations in Real CSI Samples

To examine signed relations in real CSI-Bench observations, we computed the graph shared by the signal-layer propagation modules at each stage across all test-set observations using the seed 42 checkpoint. This graph is built from motion-reference features and is distinct from non-negative learned attention. Node trajectories are centered and correlated; self-relations are excluded, the 12 strongest absolute correlations per node are retained, and each row is normalized by its absolute weight sum.

Table 7 reports the median and interquartile range of the negative-edge fraction across all observations in each test set. Figure 11 shows representative examples of the 48-node graphs and links marked edges to the corresponding feature curves.

In Table 7, the median first-stage negative-edge fraction is 23.8% for ID and rises to 26.7%, 34.2%, and 28.1% for the three cross-domain protocols. The cross-environment protocol has the highest median, which may be associated with environment changes altering static multipath; however, differences in class and user composition across protocols could also contribute. Negative-edge fractions decrease through the three stages across all protocols, and both signs remain present at every stage.

Figure 11b,c shows representative adjacency matrices; panels (d,e) link two marked edges in (b) to the corresponding feature curves. Nodes 0 and 17 tend to vary together and have a positive edge, whereas nodes 0 and 27 tend to vary in opposite directions and have a negative edge.

These test-set-wide statistics and illustrative examples connect edge signs to feature changes in real CSI samples and complement the controlled multipath study. They support interpreting signs as statistical relations, without assigning each edge to a specific propagation path.

### 4.7. Computational Cost

CUDA measurements in Table 8 use batch size 64, where per-sample times are amortized over the batch. Table 9 reports batch-size-one latency for single-observation use. The complete model contains 15.47 million parameters and processes 494 observations per second at batch 64. The graph path requires 1.890 ms per observation because it constructs sample-conditioned intra- and inter-resolution relations, while the response encoder requires 0.129 ms.

The graph encoder accounts for approximately 93% of the measured inference time. The times in Table 8 are GPU forward-pass times per sample averaged over batches of 64; CSI acquisition and host-to-device transfer are outside the timed region.

#### Recognition Performance, Computational Cost, and Application Feasibility

Table 9 compares recognition performance, parameter counts, FLOPs, inference latency, and peak GPU memory for the complete model, LSTM, Transformer, ResNet-18, and source-only CORAL. All methods are measured on the same NVIDIA GeForce RTX 4090 D using PyTorch 2.11.0+cu128, bfloat16 autocast, and FP32 stored weights. Each setting uses 30 warm-up passes and 100 CUDA-event measurements. Forward timing includes internal preprocessing but excludes acquisition, disk access, and host-to-device transfer. Peak allocated memory includes the model and input; parameter counts cover the stored inference model. GFLOPs count convolutional, linear, attention, and recurrent matrix operations, with two operations per multiply–accumulate; elementwise operations, normalization, interpolation, softmax, top-*k*, reductions, and memory operations are excluded. These measurements were collected separately from the measurements of individual modules at batch size 64 in Table 8.

The complete model achieves a mean OOD weighted-F1 of 48.81%, exceeding LSTM, Transformer, CORAL, and ResNet-18 by 4.84, 5.93, 13.69, and 10.09 percentage points, respectively. The complete model also requires more parameters, computation, and GPU memory than all four baselines. Compared with ResNet-18, the complete model uses 1.38 times the parameters and 4.33 times the counted operations. The median inference time is 3.30 ms longer than that of LSTM and approximately 11.3 times that of Transformer, showing the additional processing cost associated with the recognition gains.

CSI-Bench segments the multitask data, including HAR, into non-overlapping 5 s observations [2]. At batch size one, the complete model has median and 95th-percentile inference times of 18.93 and 35.56 ms and peak allocated GPU memory of 128.6 MiB. These times correspond to 0.38% and 0.71% of the observation duration and describe processing after the window data become available. For one prediction per observation window, these measurements support computational feasibility on the GPU used for evaluation. For overlapping windows, the processing load should be assessed against the interval between predictions.

At batch size 64, median batch latency is 122.69 ms, corresponding to approximately 522 windows/s, with peak allocated memory of 3840.9 MiB. The resulting 1.92 ms per observation characterizes batch throughput; the measurements at batch size one describe the inference delay for an individual request.

## 5. Discussion

The proposed model combines temporal changes in CSI with signed relations among subcarriers for activity recognition across acquisition conditions. The recognition experiments show differences in performance across activities, and the controlled complex-channel study supports the physical interpretation of signed relations. Measurements with prescribed motion and propagation geometry could further test this interpretation. These findings motivate considering how recognition performance could be improved and how the computational requirements affect deployment.

### 5.1. Recognition Performance and Future Improvements

**Figure 4:**
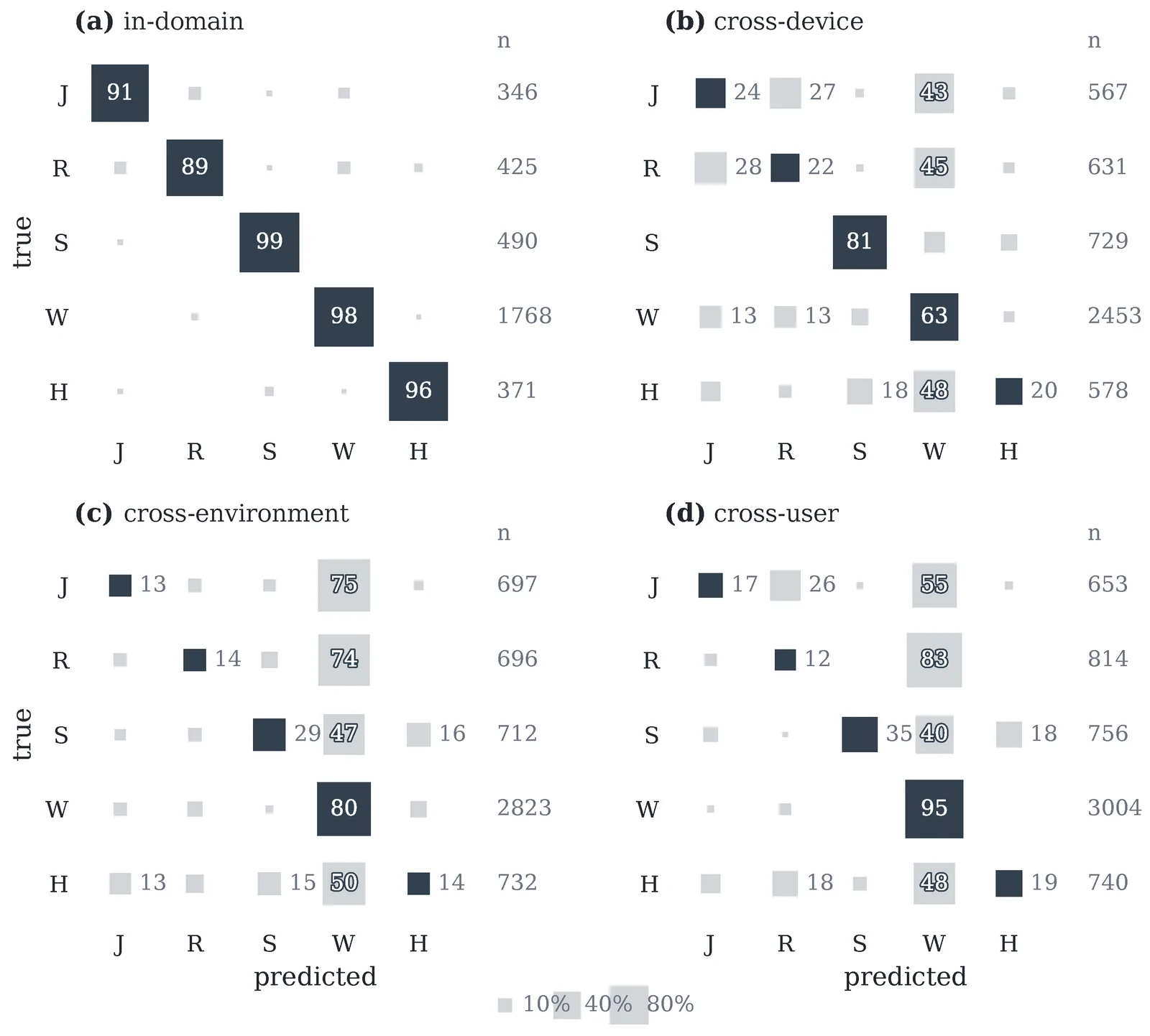
Confusion structure on the four official test protocols. Cells show row-normalized percentages of the confusion matrices aggregated over the three seeds; values of at least 10% are annotated. Classes are ordered J, R, S, W, and H (jumping, running, seated breathing, walking, and hand waving). The right margin lists true-class support *n*. The four subfigures correspond to (**a**) in-domain, (**b**) cross-device, (**c**) cross-environment, and (**d**) cross-user evaluation.

**Figure 5:**
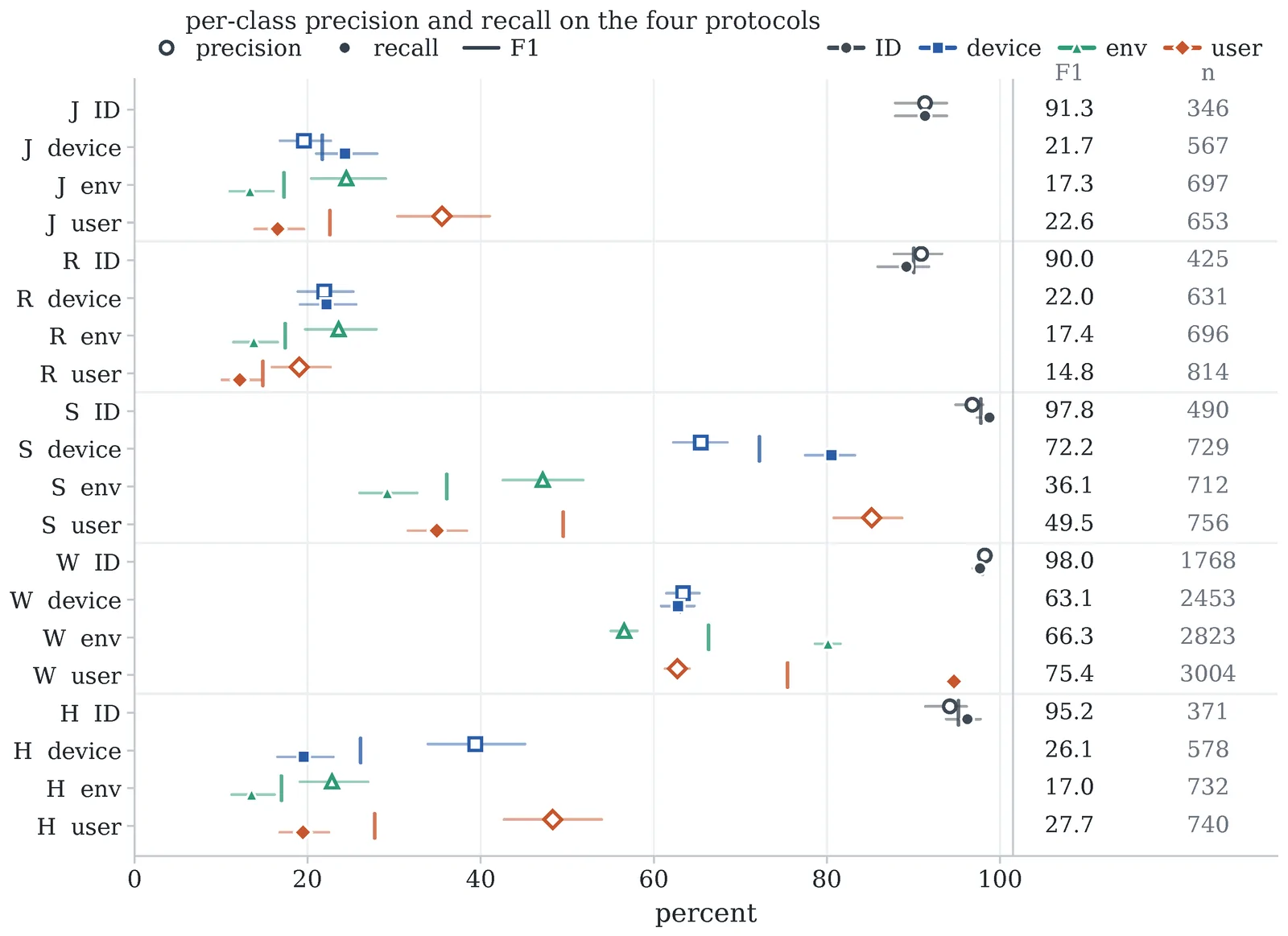
Per-class precision, recall, and F1 on the four official test protocols, computed from the confusion matrices in Figure 4.

**Figure 6:**
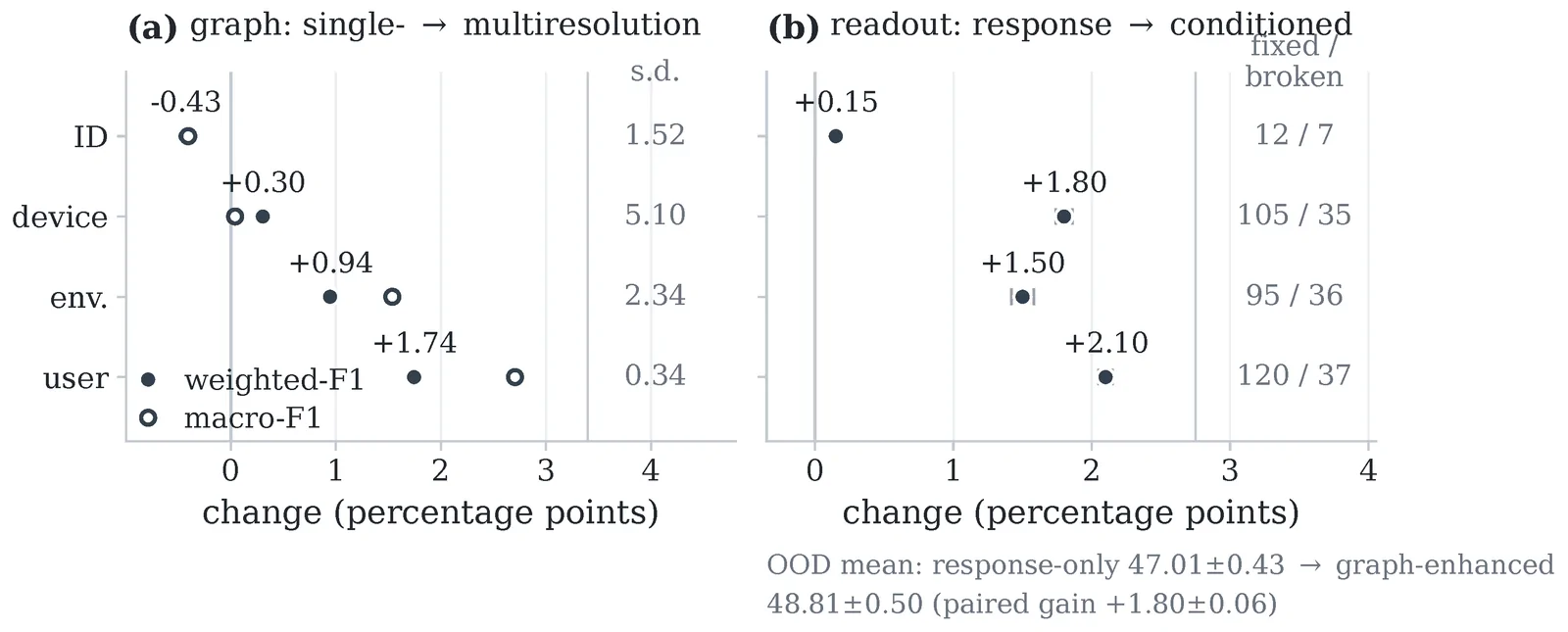
Component-change summary. (**a**) Graph-path comparison between single-resolution and multiresolution processing. (**b**) Paired change from response-only to graph-enhanced performance; error bars show the three-seed standard deviation, and the right column lists corrected/regressed counts, labeled fixed/broken in the figure.

**Figure 7:**
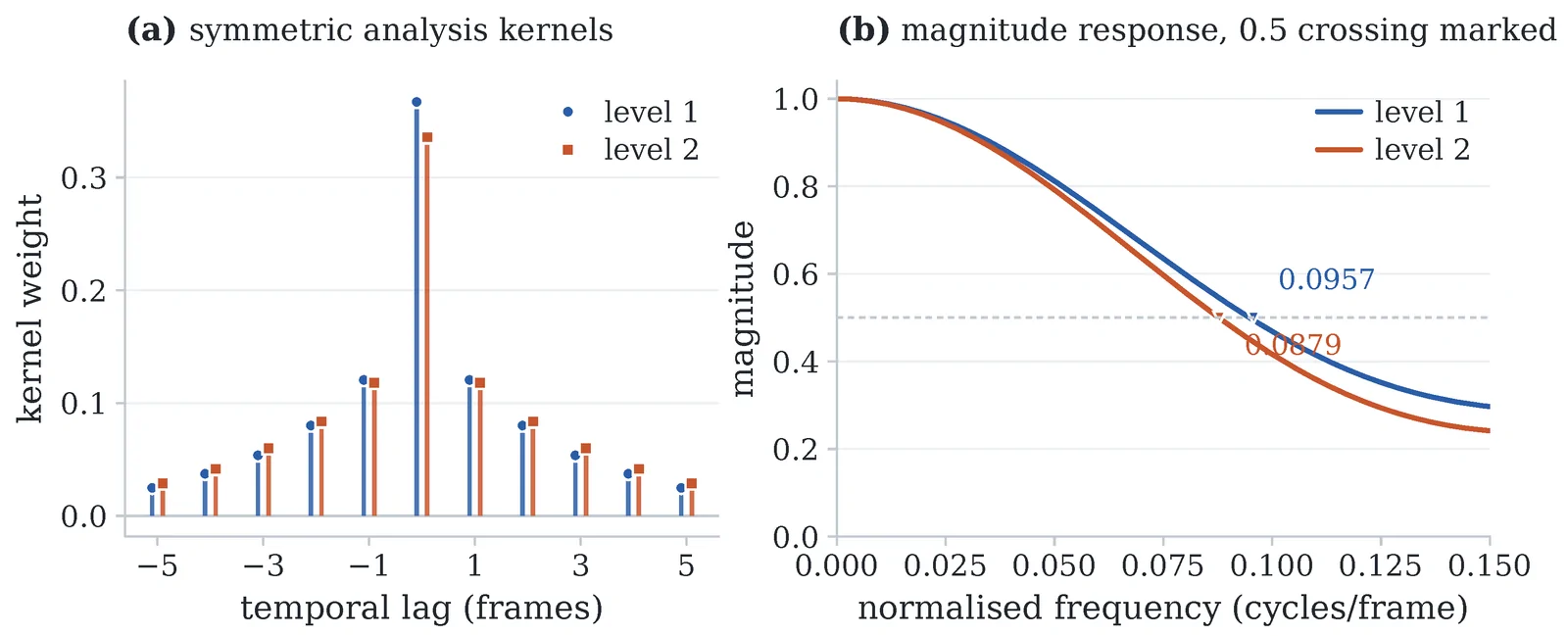
Learned multiresolution temporal analysis: symmetric smoothing kernels and their magnitude responses. Triangles mark the 0.5-magnitude crossings of the two levels.

**Figure 8:**
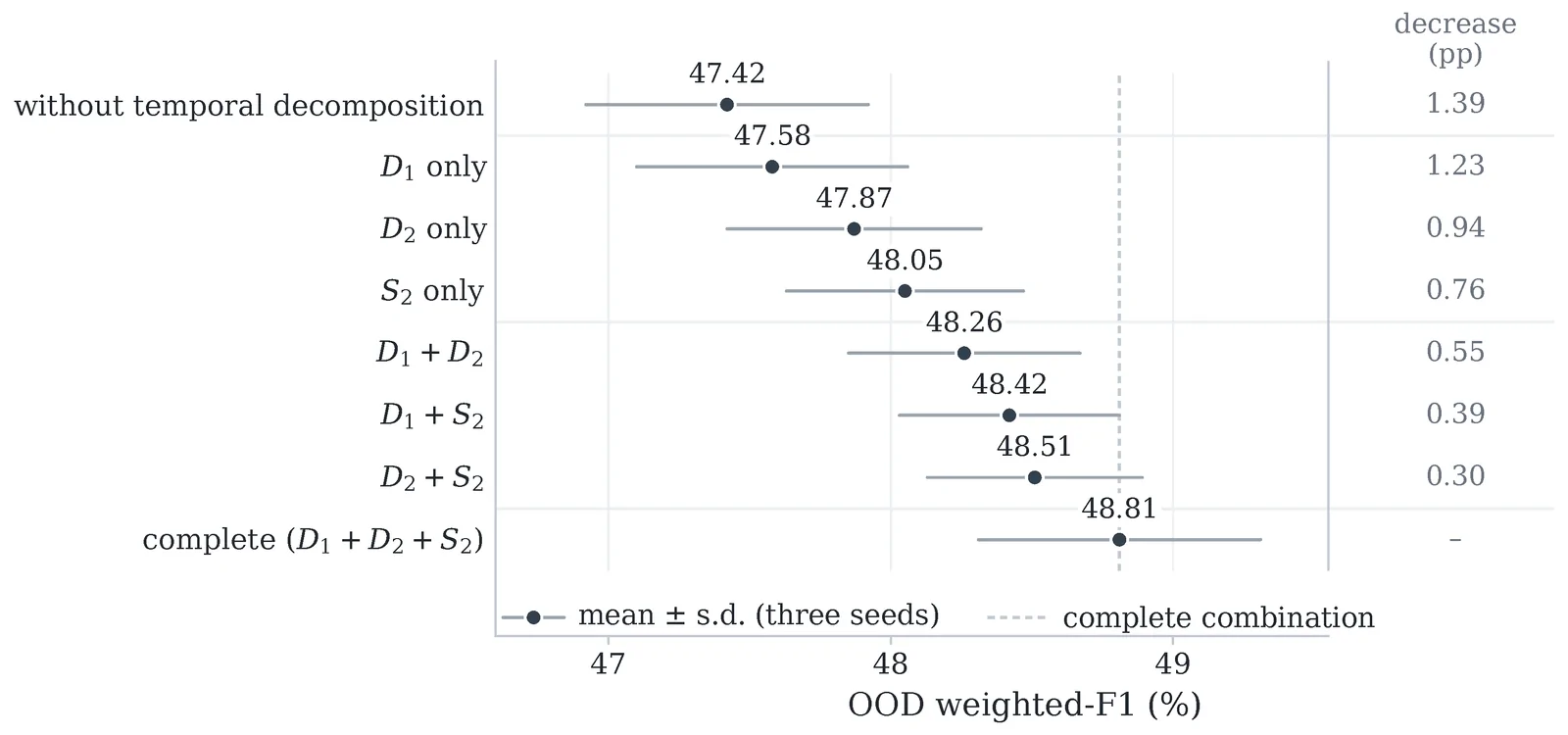
OOD weighted-F1 for different combinations of temporal components (Table 6). The dashed line marks the score of the complete combination. All configurations retain the motion reference and the fusion of graph and response features.

**Figure 9:**
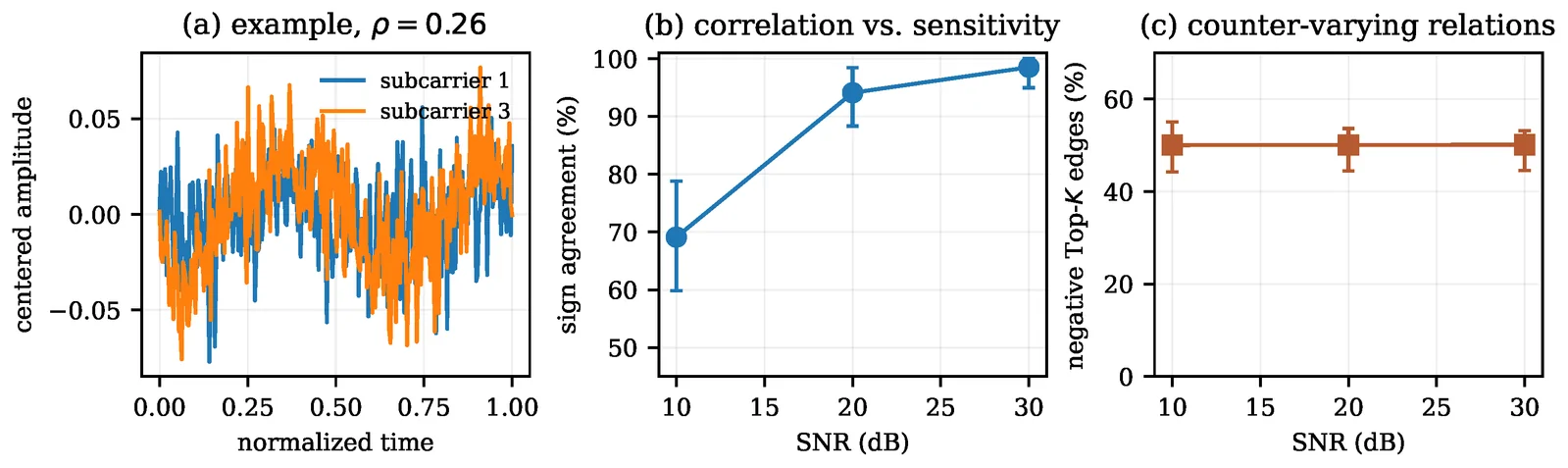
Controlled complex-multipath study with 512 independently sampled channel realizations, 48 subcarriers, 256 time steps, and K=12 neighbors per node. (**a**) Example amplitude changes from three subcarriers at 20 dB SNR. (**b**) Agreement between empirical correlation signs and analytic amplitude-sensitivity signs; error bars show the empirical 2.5th–97.5th percentile range across realizations. (**c**) Fraction of negative relations among the selected edges. In (**b**), the blue dots denote the mean sign-agreement rate at each SNR, and in (**c**), the brown squares denote the mean fraction of negative relations among the selected edges.

**Figure 10:**
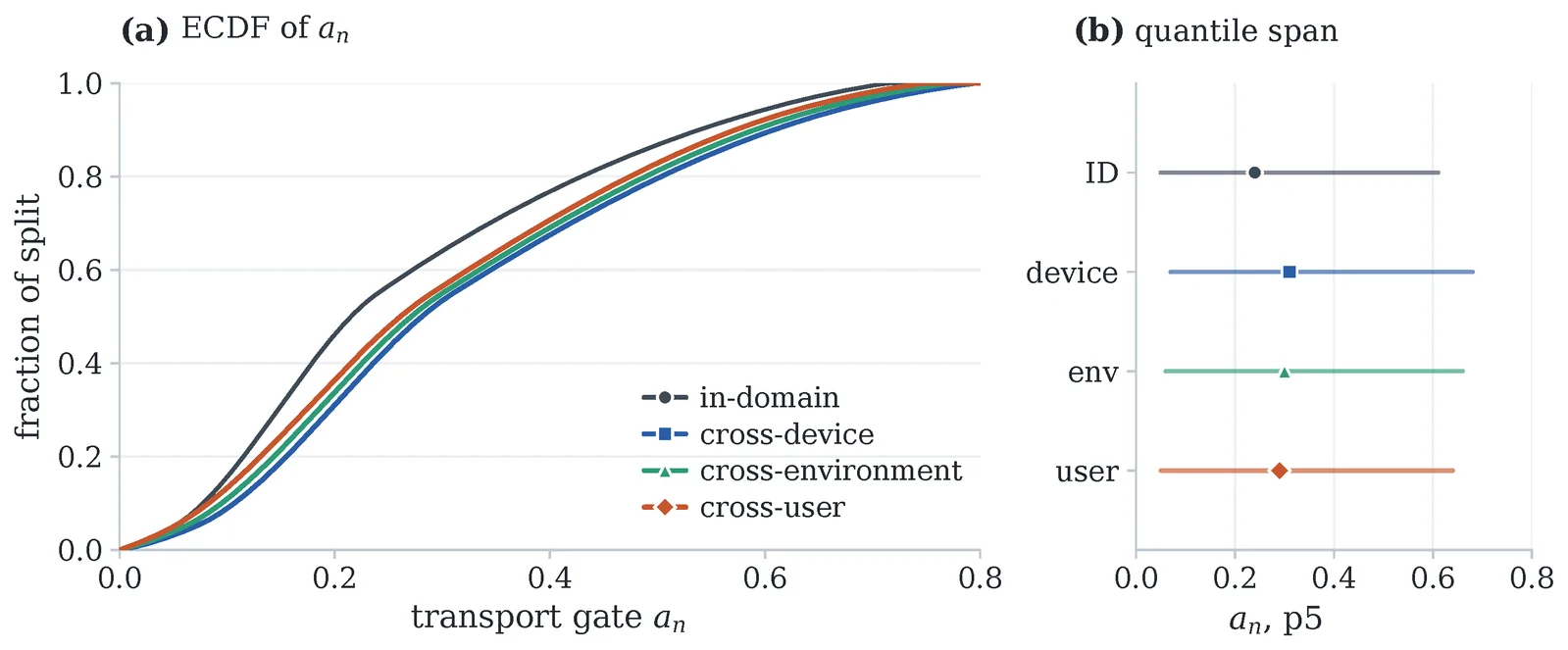
Sample-conditioned graph-to-response gate $a_{n}$. (**a**) Empirical distributions of the gate values recorded at inference on each test protocol. (**b**) 5th-to-95th percentile spans with the means marked.

**Figure 11:**
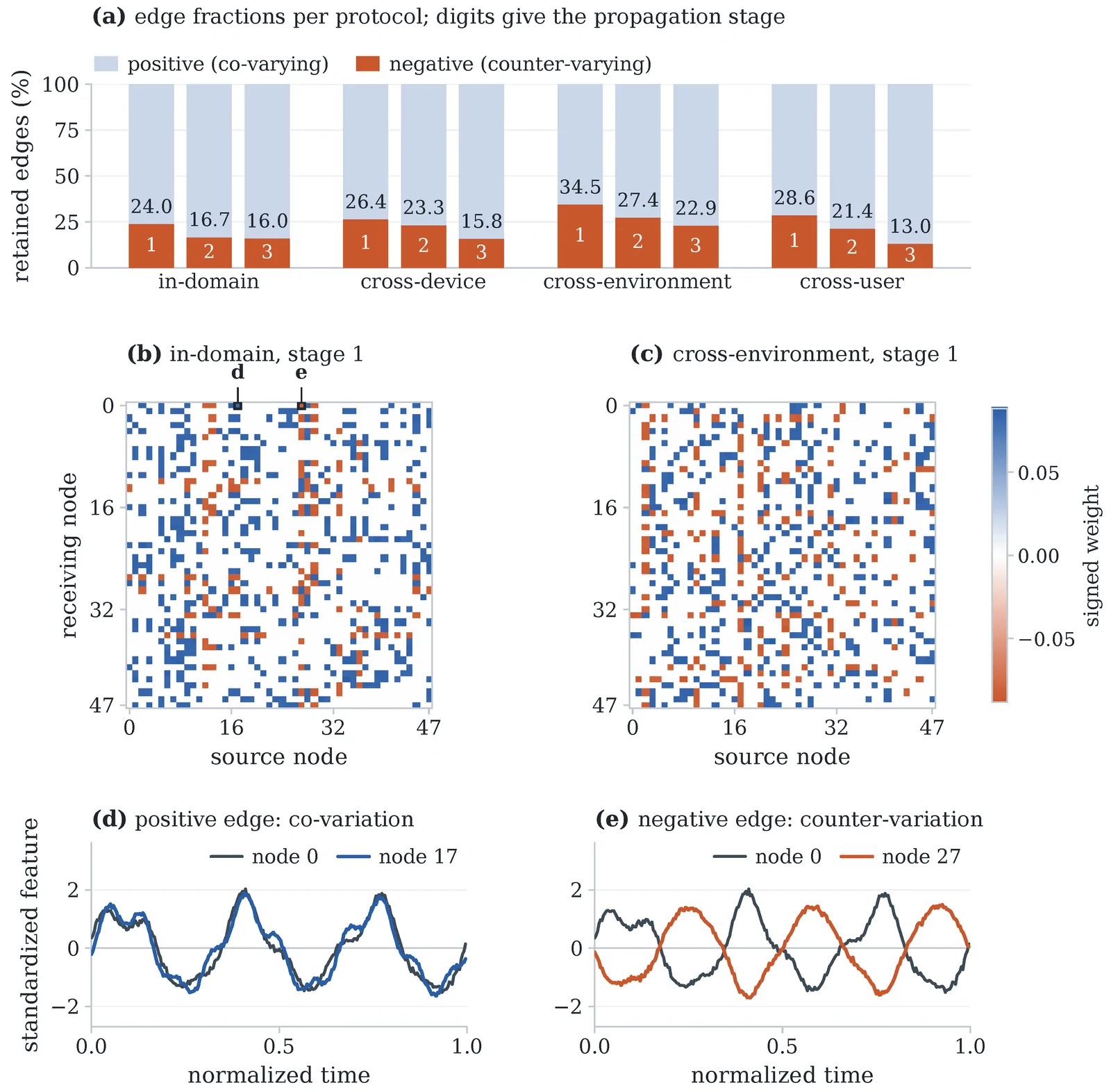
(**a**) Positive and negative edge fractions for representative samples (seed 42 checkpoint), one per protocol. (**b**,**c**) Adjacency matrices at the first stage for the ID and cross-environment examples, with a shared signed color scale; white entries are zero. Each row retains 12 neighbors; row normalization can produce asymmetry. (**d**,**e**) Standardized feature curves for the marked edges in (**b**). Pairs are selected from the first row containing both signs, using the middle node index among the retained neighbors of each sign.

The model recognizes seated breathing and walking more reliably than jumping, running, and hand waving when the device, environment, or user changes. The misclassification of jumping and running as walking in Figure 4 motivates considering velocity profiles or Doppler features that could provide clearer information about movement speed, periodicity, and brief bursts of movement. The relationship between changes in CSI and human movement speed provides a physical basis for this direction [9]. Recent studies further support using Doppler features extracted from CSI phase for activity recognition across environments and people [29,41].

These features could complement the changes in CSI over time and relationships among subcarriers at different temporal resolutions already captured by the model. Related temporal modeling approaches combine local and global features in agricultural sensor analysis [18] and separate trend and seasonal components in forecasting [20]. A future extension could add a branch for extracting velocity profiles or Doppler features and combine the output with features learned by the response and graph encoders. The benefit of adding this branch could be evaluated by comparing predictions with and without the branch under the same four protocols, focusing on whether fewer jumping and running samples are predicted as walking. Extracting Doppler features from CSI phase would require appropriately calibrated complex CSI and sampling information.

### 5.2. Computational Efficiency and Deployment Feasibility

The inference cost in Table 8 makes the graph encoder the main target for computational optimization. The implementation already uses top-*k* neighbor selection (k=12) but computes edge weights and propagates messages using dense matrix operations. Consequently, setting additional edge weights to zero leaves the matrix sizes unchanged. Potential optimizations include reducing the number of graph nodes or feature channels and using sparse matrix operations over retained edges. Khan et al. [42] demonstrate how adapting sparse matrix multiplication to the graph input and GPU architecture improves the execution of pruned graph neural networks. For the present model, these options would need to be evaluated for recognition performance, latency, and memory use while preserving informative positive and negative edges.

Deployment feasibility would need to be assessed using latency and peak memory when processing one sample at a time on the intended edge device. Recent benchmarks of graph neural networks on edge GPUs show that batch size, numerical precision, and power settings affect execution efficiency [43]. A study of sugarcane leaf disease recognition also reports inference speed and memory use alongside accuracy [19].

## 6. Conclusions

This work presents a response-conditioned multiresolution multiplex graph for Wi-Fi CSI activity sensing. Spectral alignment and channel-response encoding establish a common representation across heterogeneous subcarrier support, while constrained temporal analysis and signed multiplex propagation model motion relations within and across resolutions. Gated conditioning integrates both representations for classification. In the CSI-Bench evaluation, the model obtains 95.96%, 50.16%, 44.08%, and 52.19% weighted-F1 under ID, cross-device, cross-environment, and cross-user evaluation, with an OOD mean of 48.81%, exceeding the strongest of ten locally reevaluated baselines by 4.84 points. The separate controlled complex-multipath study tests the physical plausibility of signed amplitude correlations.

## Figures and Tables

**Table 1:** Official CSI-Bench HAR partitions used to evaluate all methods. The numbers of devices, environments, and users are computed from the sample metadata.

Partition	Samples	Classes	Devices	Environments	Users
Train ID	15,843	5	8	5	5
Validation ID	3393	5	8	5	5
Test ID	3400	5	8	5	5
Test cross-device	4958	5	2	5	5
Test cross-environment	5660	5	11	1	1
Test cross-user	5967	5	8	1	1

**Table 2:** Weighted-F1 (%) under common CSI-Bench evaluation conditions. Bold entries denote the column headers and the proposed complete model.

Model	ID	Device	Env.	User	OOD
LSTM	84.52±0.61	$\underline{42.37\pm 1.65}$	$\underline{39.83\pm 1.47}$	49.72±0.90	$\underline{43.97\pm 1.18}$
MLP	86.18±0.53	37.94±1.82	35.27±1.48	44.85±0.91	39.35±1.25
PatchTST	88.65±0.43	36.42±2.13	36.87±1.60	48.17±0.88	40.49±1.40
ResNet-18	91.42±0.27	34.85±1.51	36.57±0.76	44.73±0.67	38.72±0.90
TimeSformer-1D	86.73±0.50	39.28±1.88	37.96±1.41	49.13±0.83	42.12±1.24
Transformer	89.65±0.54	39.17±2.01	38.95±1.32	$\underline{50.52\pm 0.78}$	42.88±1.21
ViT	88.47±0.45	36.58±1.97	36.48±1.52	47.63±0.90	40.23±1.30
CORAL (source only)	92.15±0.42	27.32±2.93	33.95±1.12	44.08±0.74	35.12±1.52
DANN	91.38±0.35	31.47±2.25	34.82±1.21	44.35±0.72	36.88±1.28
SWAD	$\underline{92.17\pm 0.31}$	33.78±1.98	33.87±1.27	44.72±0.71	37.46±1.19
**Complete model**	95.96±0.08	50.16±0.44	44.08±0.46	52.19±0.59	48.81±0.50

F1 values are percentages. All rows report three-seed means and sample standard deviations. The first seven rows are locally reevaluated CSI-Bench official architectures; the next three are local domain-adaptation and domain-generalization baselines. The strongest baseline in each column is underlined.

**Table 3:** Measured (M) response-only and graph-enhanced weighted-F1 means ± sample standard deviations over three seeds on CSI-Bench. OOD is the equal-weight mean over the three cross-domain protocols. “(M)” denotes measured values from the trained checkpoints.

Configuration	ID	Cross-Device	Cross-Env.	Cross-User	OOD Mean
Response-only (M)	95.81 ± 0.08	48.36 ± 0.38	42.58 ± 0.38	50.09 ± 0.54	47.01 ± 0.43
Graph-enhanced (M)	95.96 ± 0.08	50.16 ± 0.44	44.08 ± 0.46	52.19 ± 0.59	48.81 ± 0.50

**Table 5:** Component ablation results. Values are mean OOD weighted-F1 scores over three random seeds. Differences are the decreases relative to the complete model, in percentage points. Bold entries denote the column headers and the proposed complete model.

Configuration	OOD Weighted-F1 (%)	Decrease (pp)
**Complete model**	**48.81 ± 0.50**	–
Without spectral resampling	46.99 ± 0.45	1.82
Without coordinate alignment	48.35 ± 0.35	0.46
Without multiresolution decomposition	47.42 ± 0.50	1.39
Without added signed propagation	47.96 ± 0.43	0.85
Without inter-resolution coupling	48.13 ± 0.38	0.68
Without gated conditioning	48.37 ± 0.35	0.44

OOD denotes the equal-weight mean over the cross-device, cross-environment, and cross-user protocols. Values are three-seed means ± sample standard deviations. Differences are conditional on the other components remaining in the complete model and cannot be summed into a total contribution.

**Table 6:** Recognition results for different combinations of temporal components. Values are OOD weighted-F1 scores. Differences are the decreases relative to the complete combination, in percentage points. Bold entries denote the column headers and the complete combination.

Temporal Components	OOD Weighted-F1 (%)	Decrease (pp)
Input without temporal decomposition	47.42 ± 0.50	1.39
$D_{1}$ only	47.58 ± 0.48	1.23
$D_{2}$ only	47.87 ± 0.45	0.94
$S_{2}$ only	48.05 ± 0.42	0.76
$D_{1}+D_{2}$	48.26 ± 0.41	0.55
$D_{1}+S_{2}$	48.42 ± 0.39	0.39
$D_{2}+S_{2}$	48.51 ± 0.38	0.30
**Complete: $D_{1}+D_{2}+S_{2}$**	**48.81 ± 0.50**	–

OOD is the equal-weight mean over the three cross-domain protocols. Values are three-seed means ± sample standard deviations.

**Table 7:** Test-set negative-edge fraction statistics by protocol and propagation stage. Each observation produces a graph with 576 retained nonzero directed edges; the table reports medians and interquartile ranges over all test observations in each protocol.

Protocol	Negative-Edge Fraction (%), Median [Q1–Q3]		
	Stage 1	Stage 2	Stage 3
ID	23.8 [20.9–26.7]	17.1 [14.3–19.8]	15.7 [13.2–18.3]
Cross-device	26.7 [22.1–31.3]	22.8 [18.7–27.1]	16.2 [12.8–19.4]
Cross-environment	34.2 [28.8–39.4]	27.9 [23.4–32.6]	22.4 [18.1–26.8]
Cross-user	28.1 [24.0–32.6]	21.8 [17.9–25.6]	13.4 [10.3–16.7]

Each cell reports the median and interquartile range [Q1–Q3] of the negative-edge fraction across all observations in the test set. Each observation produces a 48-node graph with 576 retained nonzero directed edges.

**Table 8:** Model size and CUDA inference cost at batch size 64 using bfloat16 on an NVIDIA GeForce RTX 4090 D. Bold entries denote the column headers.

Module	Parameters (M)	ms/Sample	Samples/s	Peak Memory (MiB)
Channel-response encoder	11.48	0.129	7739	599
Multiresolution graph encoder	2.94	1.890	529	3845
Relational conditioning	0.92	–	–	–
Complete model	15.47	2.025	494	3845

**Table 9:** Measured recognition performance and computational cost under common evaluation conditions. OOD weighted-F1 is reported as the mean ± standard deviation across three random seeds. Latency and peak memory use batch size one; P95 is the 95th percentile of the 100 inference timings. Bold entries denote the column headers and the proposed complete model.

Model	OOD Weighted-F1	Parameters	GFLOPs	Latency (ms)		Peak Memory
	(%)	(M)		Median	P95	(MiB)
LSTM	43.97 ± 1.18	2.828	2.687	15.63	17.48	68.5
Transformer	42.88 ± 1.21	3.367	4.244	1.67	2.09	31.6
ResNet-18	38.72 ± 0.90	11.179	3.627	2.06	4.85	67.6
CORAL (source-only)	35.12 ± 1.52	3.367	4.244	1.69	1.97	31.6
**Complete model**	**48.81 ± 0.50**	15.469	15.717	18.93	35.56	128.6

OOD is the equal-weight mean over the three cross-domain protocols. Parameter and operation counts, forward timings, memory, and F1 values are obtained from the evaluated implementations; the operation coverage is defined in the text.

## Data Availability

CSI-Bench is available from the dataset repository cited in [2].
